# HuR expression in adipose tissue mediates energy expenditure and acute thermogenesis independent of UCP1 expression

**DOI:** 10.1080/21623945.2020.1782021

**Published:** 2020-07-25

**Authors:** Sarah R. Anthony, Adrienne Guarnieri, Lindsey Lanzillotta, Anamarie Gozdiff, Lisa C. Green, Katherine O’Grady, Robert N. Helsley, A. Phillip Owens, Michael Tranter

**Affiliations:** a Department of Internal Medicine, Division of Cardiovascular Health and Disease, University of Cincinnati College of Medicine, Cincinnati, OH, USA; b Division of Pediatrics, Department of Gastroenterology, Hepatology, and Nutrition, University of Kentucky College of Medicine and Kentucky Children’s Hospital, Lexington, KY, USA

**Keywords:** HuR, adipose tissue, brown fat, thermogenesis, UCP1, gene expression, calcium transport

## Abstract

The goal of this study was to define the functional role of adipocyte-specific expression of the RNA binding protein Human antigen R (HuR). Mice with an adipocyte-specific deletion of HuR (*Adipo-HuR^−/-^*) were generated by crossing HuR floxed (*HuR^fl/fl^*) mice with mice expressing adiponectin-driven cre-recombinase (*Adipoq-cre*). Our results show that *Adipo-HuR^−/-^* mice display a lean phenotype compared to wild-type littermate controls. HuR deletion results in a diet-independent reduction in percent body fat composition along with an increase in energy expenditure. Functionally, *Adipo-HuR^−/-^* mice show a significant impairment in acute adaptive thermogenesis (six hours at 4°C), but uncoupling protein 1 (UCP1) protein expression in brown adipose tissue (BAT) is unchanged compared to control. Pharmacological inhibition of HuR also results in a marked decline in core body temperature following acute cold challenge independent of UCP1 protein expression. Among the 588 HuR-dependent genes in BAT identified by RNA-seq analysis, gene ontology analysis shows a significant enrichment in mediators of calcium transport and signalling, almost all of which are decreased in *Adipo-HuR^−/-^* mice compared to control. In conclusion, adipocyte expression of HuR plays a central role in metabolic homoeostasis and mediates UCP1-independent thermogenesis in BAT, potentially through post-transcriptional control of intracellular calcium transport.Abbreviations: Adipo-HuR^−/-^: Adipocyte-specific HuR deletion mice; BAT: Brown adipose tissue; HuR: Human antigen R; UCP1: Uncoupling protein 1

## Introduction

Obesity, a heterogeneous metabolic disease characterized by an excessive accumulation of body fat, is a rising economic burden in the United States as well as an obstacle to individual and societal health and longevity. Strong evidence has provided a link between obesity and several chronic diseases such as type II diabetes, respiratory disease, vascular disease, cancer, and cardiovascular disease. There is a current unmet need for effective obesity therapeutics, and stimulating energy expenditure in mitochondria-rich brown adipose tissue (BAT) represents a promising strategy/therapeutic target for safely increasing metabolic rate and reducing obesity.

Unlike white adipose tissue (WAT), the primary role of which is to store energy in the form of lipid droplets, BAT is highly metabolically active, uncoupling mitochondrial ATP synthesis resulting in the generation of heat. Recent work has demonstrated the existence of metabolically active BAT in adult humans [1–3]. More importantly, the energy expenditure of human BAT can be enhanced through cold or adrenergic stimulation, similar to BAT in rodents [1,3–6], suggesting these processes could be manipulated for therapeutic benefit. The thermogenic uncoupling of mitochondrial ATP synthesis, which can account for up to 20% of total energy expenditure [7], is achieved by the uncoupling proteins (UCP). There are three distinct (and conserved) members of the UCP family found in the human genome: UCP1, UCP2, and UCP3. UCP1, expressed exclusively in BAT, is upregulated by cold-induced β_3_-adrenergic signalling, and is therefore critically important for the regulation of BAT-mediated thermogenesis [6,8].

Human antigen R (HuR; ELAV-like protein 1 – *elavl1*) is an RNA binding protein that mediates gene expression through stabilization of mRNA targets and is highly expressed in both WAT and BAT [9]. Prior *in vitro* evidence shows that HuR protein expression is increased during adipocyte differentiation in 3T3-L1 cells, and siRNA-mediated knockdown of HuR inhibits adipocyte differentiation via decreased C/EBPβ, a key transcriptional regulator of peroxisome proliferator-activated receptor gamma (PPAR-γ) [10,11]. HuR has also been suggested to regulate the expression of a critical glucose transporter (GLUT-1) in mature adipocytes [12]. Despite this compelling *in vitro* data, a clear role for HuR in adipocyte function *in vivo* has yet to be delineated. To determine a functional role for HuR in adipocyte biology, we generated adipocyte-specific HuR-deficient mice (*Adipo-HuR^−/-^*) and show that deletion of HuR leads to a disruption of BAT architecture and acute thermogenic function independent of UCP1 expression. Application of RNA-seq analysis to identify the underlying mechanisms via transcriptome-wide changes in HuR-dependent gene networks show a suppression of genes involved in intracellular calcium cycling upon HuR deletion.

## Materials and methods

### Mouse models

All mouse studies were approved by the University of Cincinnati Institutional Animal Care and Use Committee (IACUC). HuR floxed mice were described by Ghosh et al [13] and obtained from Jackson Labs (stock # 021431). To generate an adipose-specific HuR deletion model (*Adipo-HuR^−/-^*), HuR-floxed mice were crossed with mice harbouring an adiponectin-specific Cre recombinase transgene (*AdipoQ-Cre*) (Jackson Labs, stock # 010803), a gift from Dr. David Hui (University of Cincinnati). Wild-type (*wt/wt-cre^+^; flox/flox-cre^−^; wt/flox-cre^−^*) littermates were used as controls. Mice were housed in the University of Cincinnati vivarium at 23°C.

### Metabolic studies

Male mice, 9 weeks of age, were placed on a high fat diet (HFD; 45% kcal from fat; Research Diets, Inc, D12451) or control chow (10% kcal from fat; D12450B) for 22 weeks. Body weight was measured weekly. Glucose tolerance was assessed after 20 weeks on diet by continuous monitoring of blood glucose levels following an intraperitoneal injection of 45% glucose solution (1 g/kg). Total food intake, activity, oxygen consumption (vO_2_), and carbon dioxide (vCO_2_) production were continuously measured for individual mice over three consecutive days via indirect calorimetry using a Comprehensive Lab Animal Monitoring System (CLAMS/Oxymax, Columbus Instruments) after 21 weeks on diet. Data shown is based on the three-day average for each mouse. NMR was used to determine percent lean and fat body mass. Mice were euthanized after 22 weeks on diet. All metabolic measurements and body mass composition analyses were performed by the University of Cincinnati Mouse Metabolic Phenotyping Core (MMPC).

### Tissue and plasma processing

At time of euthanasia, mice were sedated with 3% isoflurane and blood collected into 3.2% sodium citrate to collect plasma via centrifugation at 4,000 x g at room temperature, which was snap frozen. Mice were subsequently euthanized via thoracotomy and tissues were removed, weighed, and either flash frozen in liquid nitrogen or fixed in 4% paraformaldehyde in PBS for further analysis. Plasma lipid assessments (total cholesterol, nonesterified fatty acids (NEFAs), and triglycerides) were performed via colorimetric assays at the MMPC.

### Cold challenge

Mice were fasted overnight, placed in individual cages with food and bedding removed at 4°C. Core body temperature was monitored by rectal probe at least every hour. When core body temperature fell outside of euthermic range (below 32°C), mice were returned to room temperature with bedding, food, and supplemental care. Thermal imaging was done using a Flir One Pro thermal camera (Flir Systems, Inc.).

### Pharmacological inhibition of HuR

Ten-week old male *C57BL/6J* mice (Jackson Labs, stock #000664) underwent six-hour cold challenge as described above, with intraperitoneal injection of HuR inhibitor (Dihydrotanshinone; 10 mg/kg; Sigma D0947) or vehicle (DMSO) one hour prior to cold exposure. Mice were euthanized immediately following cold challenge, and blood and tissues were collected, as described in the previous sections.

### Histological analysis

Fixed tissues were paraffin embedded by the Cincinnati Children’s Hospital Medical Center Department of Pathology Research Core (Cincinnati, OH, USA) and sectioned at 6 μm thickness and subsequently stained with haematoxylin and eosin (H&E). Lipid droplet size was calculated using edge thresholding in ImageJ (NIH, Bethesda, MD).

### Protein isolation and Western blotting

Total protein was isolated from crushed tissue in RIPA buffer with 0.5 mM DTT, 0.2 mM sodium-orthovanadate, and a protease inhibitor mixture tablet (Complete mini; Roche Applied Science). Ten μg of protein extract per lane was separated on a 10% polyacrylamide gel and transferred to a nitrocellulose membrane. Blocking was performed for 1 h at room temperature using 4% BSA in 0.1% Tween 20, tris-buffered saline (T-TBS). Primary antibodies for UCP1 (Novus Biologicals NBP2-20,796) and HuR (AbCam ab200342) were incubated overnight at 4°C, and secondary antibodies were incubated for 1–2 h at room temperature in T-TBS. Loading was normalized to total protein using TGX Stain Free precast gels (BioRad, Hercules, CA) as described [14]. Images were captured and analysed using a ChemiDoc Imaging System and ImageLab software (BioRad).

### RNA-seq analysis

RNA was isolated from BAT as previously described [15] and poly(A) library preparation and sequencing were done by the CCHMC DNA sequencing and genotyping core. Sequence read mapping, principal component analysis, differential expression analysis, heat mapping, and generation of volcano plots were done using CLC Genomics Workbench (v. 20.0.2, Qiagen) as previously described [16]. The statistical significance threshold for expression between groups was defined as an FDR-corrected *P*-value less than or equal to 0.05 and a fold-change greater than or equal to 1.5. Gene ontology analysis was done using the NIH DAVID Bioinformatics Functional Annotation Tool [17,18] with an EASE score/*P*-value threshold of 0.05 and a minimum of 5 genes per group as previously described [19]. All RNA-seq data is available in the NCBI Gene Expression Omnibus (https://www.ncbi.nlm.nih.gov/geo/) (GSE154820).

### Statistical analysis

Data is graphed as represented as mean ± SEM unless otherwise noted. All data was tested for normality (Shapiro-Wilk) and equal variance (Brown-Forsythe). Serial measurements were analysed using multiple comparisons within a two-way ANOVA. Cold challenge was assessed using a log-rank (Mantel-Cox) test. Individual comparisons were done using Student’s t-test. All graphs were created and statistical analyses performed using GraphPad Prism 8.

## RESULTS

### Adipocyte-specific HuR-deletion induces a lean phenotype in mice

**Figure 1.**
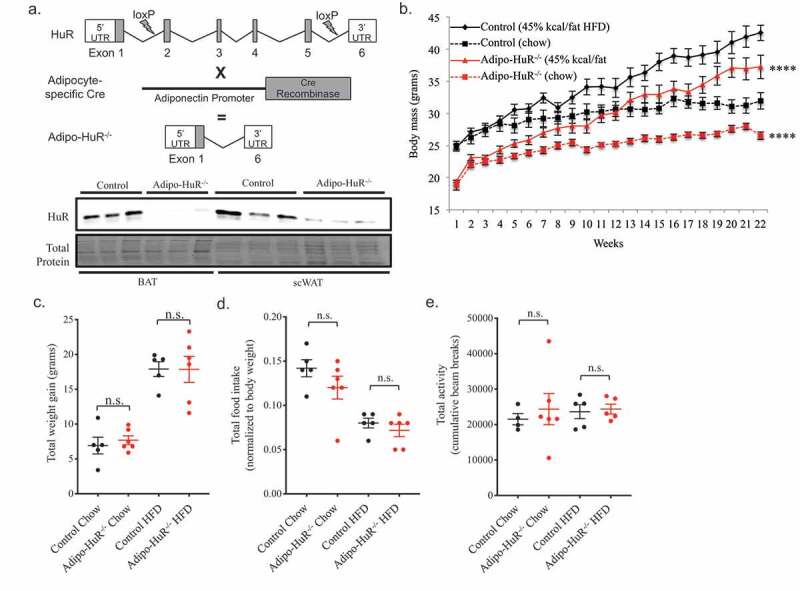
**Adipocyte-specific deletion of HuR induces a lean phenotype**. (a) Genetic scheme used to induce adipocyte-specific deletion of HuR and representative Western blot showing relative HuR expression in littermate control and *Adipo-HuR^−/-^* mice. (b) *Adipo-HuR^−/-^* and littermate control mice were placed on a 45% kcal/fat high fat or control diet for 22 weeks with weekly assessment of body weight. (c) Total weight gain in control and *Adipo-HuR^−/-^* mice following 22 weeks on 45% kcal/fat high fat or control diet. Total average daily food intake (d) and total activity (e) were assessed in each group using comprehensive lab animal monitoring systems (CLAMS) cages. For b-e, n ≥ 5 per group. **** *P* ≥ 0.0001 vs control group

**Figure 2.**
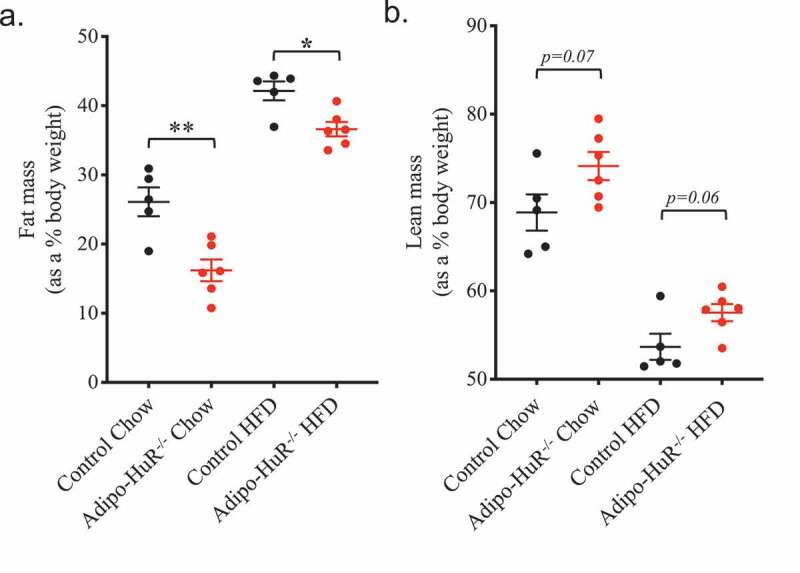
**Adipocyte-specific HuR mediates total body composition of fat and lean mass**. Body composition of (a) total fat and (b) lean mass were determined by NMR following 22 weeks on chow or HFD. n ≥ 5 per group. *P ≥ 0.05; ** *P* ≥ 0.01

Prior *in vitro* work suggested a role for HuR in adipocyte differentiation [10], but the functional role of HuR in mature adipocytes *in vivo* has not yet been explored. We crossed HuR floxed mice [13,16] with adiponectin-cre expressing mice to induce adipocyte-specific deletion of HuR (*Adipo-HuR^−/-^*) (Figure 1(a); Fig. S1). *Adipo-HuR^−/-^* mice have a lower starting body weight compared to wild-type littermate controls that is maintained following 22 weeks on HFD or chow (Figure 1(b)), with the total weight gain in *Adipo-HuR^−/-^* and control mice being equivalent following a HFD challenge (Figure 1(c)). Importantly, *Adipo-HuR^−/-^* mice have no observable phenotype that would indicate developmental abnormalities (tibia and anogenital lengths, with and without adjustment for body weight, were both observed to be unchanged from controls; Fig. S2), and have similar food intake and total activity compared to control counterparts (Figure 1(d-e)). Analysis of body composition by NMR shows a significant reduction in percent fat mass in *Adipo-HuR^−/-^* mice (Figure 2(a)) accompanied by a trend to increase the percent lean mass (Figure 2(b)).

**Figure 3.**
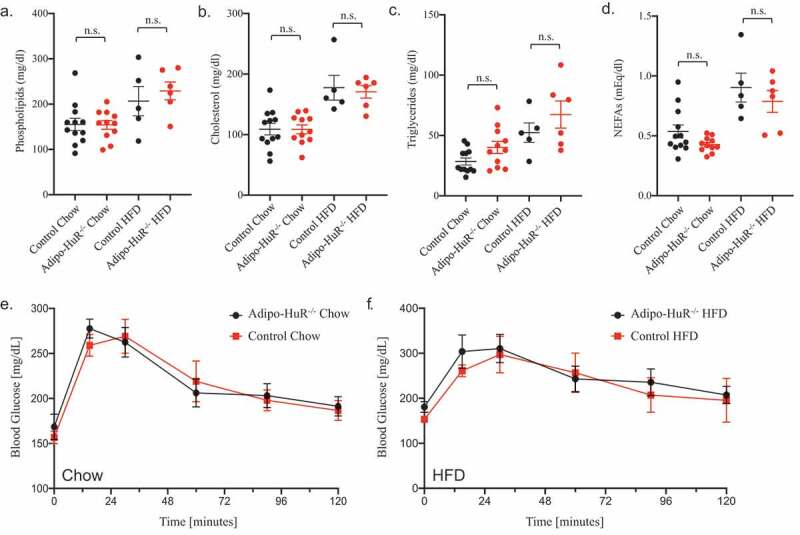
**Adipocyte-specific deletion of HuR does not affect serum lipids or glucose tolerance**. Total serum (a) phospholipids, (b) cholesterol, (c) triglycerides, and (d) non-esterified fatty acids (NEFAs) were assessed in control and *Adipo-HuR^−/-^* mice following 22 weeks on 45% kcal/fat or control diet. Glucose tolerance was assessed in mice fed control (e) or 45% kcal/fat diet (f). n ≥ 5 per group. No statistical significance was found among any groups

Interestingly, *Adipo-HuR^−/-^* mice show no difference in serum phospholipids (Figure 3(a)), cholesterol (Figure 3(b)), triglycerides (Figure 3(c)), or NEFAs (Figure 3(d)) compared to control mice on either chow or HFD. Similarly, deletion of HuR does not impact glucose tolerance in chow or HFD fed mice (Figure 3(e-f)).

### Adipocyte expression of HuR mediates whole body energy expenditure

**Figure 4.**
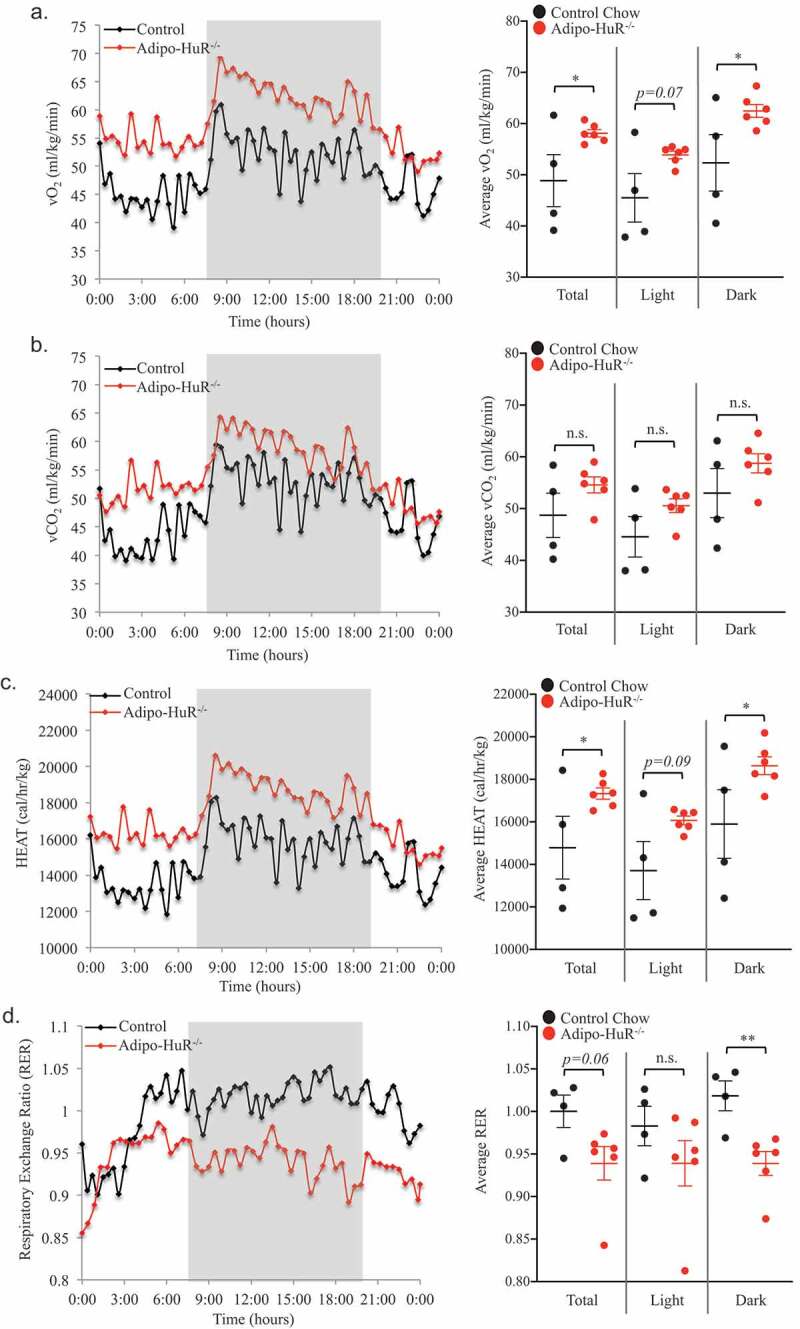
**Energy expenditure is increased in Adipo-HuR^−/-^ mice**. Daily oxygen consumption (vO_2_) (a) and vCO_2_ production (b) were measured using the Oxymax system. Total energy expenditure (HEAT) (c) and respiratory exchange ratio (RER) (d) were then determined based on vO_2_ and vCO_2_ values. Shaded area represents dark housing cycle and data is represented as both rolling daily average (left) and total, light, and dark averages (right). n ≥ 4 per group. *P ≥ 0.05; ** *P* ≥ 0.01

To determine if HuR in the adipocytes plays a functional metabolic role, energy expenditure was assessed by CLAMS. Oxygen consumption (vO_2_; ml/kg/min) and total energy expenditure (HEAT; cal/hr/kg) were both significantly increased in *Adipo-HuR^−/-^* mice (Figure 4(a,c), respectively). However, the amount of CO_2_ produced (vCO_2_; ml/kg/min) was not significantly different between the two groups (though trending higher in *Adipo-HuR^−/-^* mice; Figure 4(b)), thus leading to significant changes in the respiratory exchange ratio (RER) between the two groups (Figure 4(d)) suggestive of differential substrate utilization. These HuR-mediated changes in energy expenditure are also independent of diet as similar changes were observed in HFD fed mice (Fig. S3).

### HuR mediates individual fat depot mass and lipid droplet size in BAT

**Figure 5.**
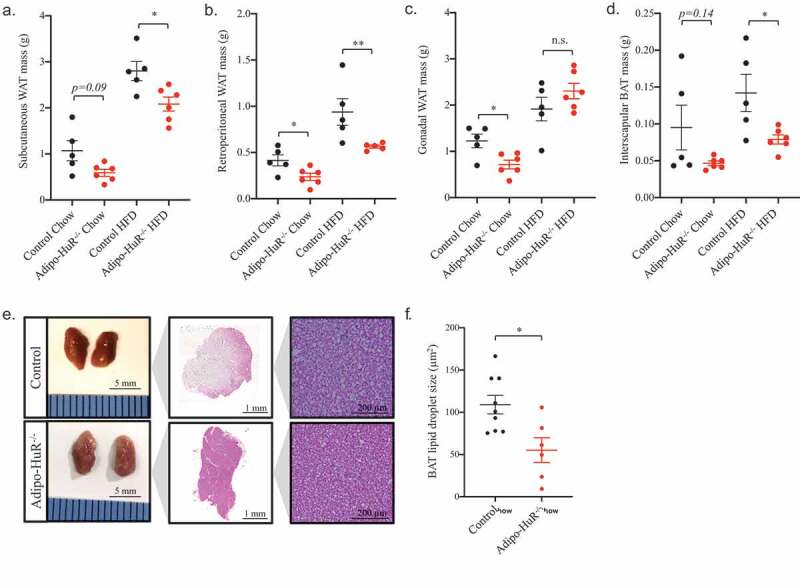
**HuR deletion reduces individual fat depot mass and BAT lipid droplet size**. Mass of subcutaneous (a), retroperitoneal (b), and gonadal (c) WAT and interscapular BAT (d) were assessed for each animal upon euthanasia. (e) Representative images of whole and H&E stained interscapular BAT from control and *Adipo-HuR^−/-^* mice. (f) Quantification of individual lipid BAT droplet size. n ≥ 5 per group. *P ≥ 0.05; ** *P* ≥ 0.01

As the primary phenotypic difference in *Adipo-HuR^−/-^* mice is percent fat mass, we investigated the impact of HuR deletion on the mass of individual adipose depots. Similar to the decrease in total percent body fat observed in Figure 2(a), *Adipo-HuR^−/-^* mice have a significant (or strongly trending) decrease in individual fat depot mass of subcutaneous, retroperitoneal, and gonadal WAT and interscapular BAT (Figure 5(a-d), respectively; similar trends were observed when normalized to body mass – data not shown). However, upon deeper examination, the most striking phenotypic observation at the cellular level was increased basal density of BAT from *Adipo-HuR^−/-^* mice maintained on a control chow diet (Figure 5(e)) accompanied by a decrease in lipid droplet size (Figure 5(f)).

### HuR mediates acute thermogenesis independent of UCP1 expression

**Figure 6.**
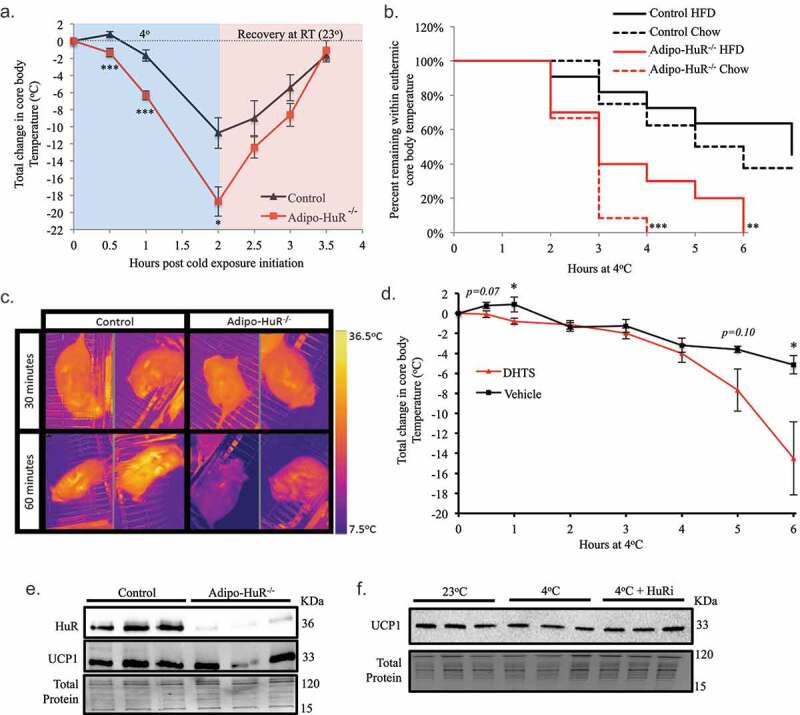
**HuR mediates acute thermogenesis independent of UCP1 expression**. (a) Control and *Adipo-HuR^−/-^* mice were subjected to a 4°C cold challenge with hourly monitoring of core body temperature. (b) Percentage of mice remaining within euthermic core body temperature range (32-37°C) at each hour. (c) Representative thermal images of control and *Adipo-HuR^−/-^* mice after 30 and 60 minutes at 4°C. (d) Mice with treated with HuR pharmacological inhibitor (DHTS; 10 mg/kg) or vehicle control and exposed to 4°C cold challenge for 6 hours. (e) Representative Western blot of HuR and UCP1 expression in BAT from control and *Adipo-HuR^−/-^* mice. (f) Representative Western blot of UCP1 protein expression in BAT from wild-type mice housed at room temperature (25°C), 4°C, or 4°C in the presence of a HuR pharmacological inhibitor (DHTS; 10 mg/kg) for 6 hours. For a and b, n ≥ 8 per group. *P ≥ 0.05; ** P ≥ 0.01; *** P ≥ 0.001

To assess whether the difference in BAT lipid droplet size has a functional impact on the thermogenic function, mice were subjected to a six-hour cold challenge at 4°C with hourly monitoring of core body temperature. *Adipo-HuR^−/-^* mice exhibited a more rapid decline and larger drop in core body temperature within the first few hours when housed at 4°C (Figure 6(a)). Additionally, *Adipo-HuR^−/-^* had a much smaller proportion of mice maintaining euthermic core body temperature following 6 hours at 4°C (Figure 6(b)), with a visual difference in body temperature via thermal imaging after just 60 minutes at 4°C (Figure 6(c)).

To determine whether the thermogenic deficiency of *Adipo-HuR^−/-^* mice is an acute effect mediated by HuR in response to cold and not a compensatory effect of HuR deletion, a separate group of *C57BL/6 J* mice were subjected to acute cold challenge following pre-treatment with a HuR pharmacological inhibitor. Compared to vehicle treated mice, mice given a HuR inhibitor (DHTS, 10 mg/kg) 60 minutes prior to cold exposure exhibit a larger drop in core body temperature (Figure 6(d)). Interestingly, HuR inhibition also prevented the immediate small increase in core body temperature in vehicle treated mice within the first hour of cold exposure (also observed in control mice in Figure 6(a)).

Because of the central role of UCP1 in BAT-mediated thermogenesis [8], we sought to determine if differences in UCP1 expression may be an underlying mechanism for the observed HuR-dependent thermogenesis. Surprisingly, our results show that *Adipo-HuR^−/-^* mice do not have significantly different levels of UCP1 protein expression at baseline compared to control mice (Figure 6(e)). In addition, UCP1 protein expression in the *C57BL/6J* mice was not altered by a six-hour cold challenge and pharmacological inhibition of HuR has no influence on UCP1 expression (Figure 6(f)). Together, these results demonstrate that HuR is necessary for acute adaptive thermogenesis in response to a cold challenge, independent of UCP1-expression.

### HuR mediates expression of calcium transport genes in BAT

**Figure 7.**
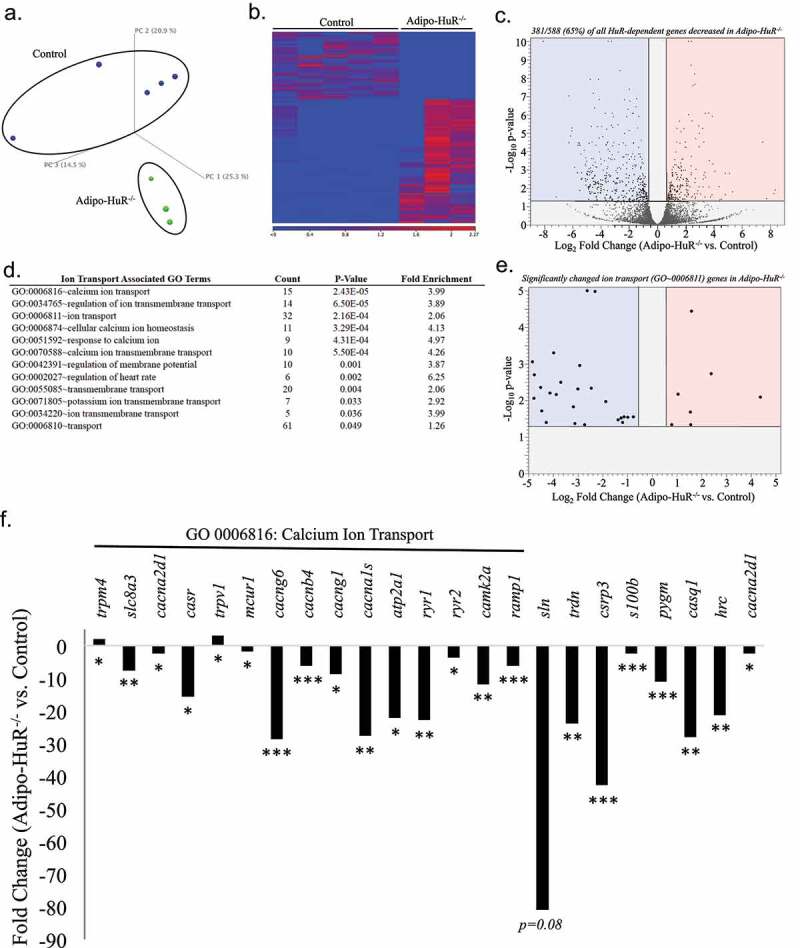
**HuR mediates expression of ion transport and calcium cycling genes in BAT**. (a) Principal component (PC) analysis of RNA-seq results from total BAT RNA from control and *Adipo-HuR^−/-^* mice. (b) Heat map of gene expression differences in BAT from control and *Adipo-HuR^−/-^* mice. (c) Volcano plot showing total fold change (log_2_; x-axis) and p-value (log_10_; y-axis) of all genes in *Adipo-HuR^−/-^* BAT relative to control BAT. Blue and red regions represent significantly down and up-regulated genes, respectively. (d) A list of all significantly enriched Ion Transport associated GO Terms among HuR-dependent gene expression in BAT. (e) Volcano plot showing total fold change and p-value of significant HuR-dependent changes among genes in the *Ion Transport* parent gene ontology. (f) HuR-dependent gene expression changes within the *Calcium Ion Transport* gene ontology (denoted above the x-axis) as well as additional significant calcium transport genes. n = 5 control BAT; n = 3 *Adipo-HuR^−/-^* BAT. *P ≥ 0.05; ** P ≥ 0.01; *** P ≥ 0.001

Since HuR primarily functions as a post-transcriptional mediator of gene expression, RNA-seq was used to identify the mechanistic effect of HuR deletion on transcriptome-wide gene expression changes in BAT (Figure 7(a-c)). Differential expression analysis identified 588 genes that were significantly changed in *Adipo-HuR^−/-^* mice compared to control (FDR P-Value ≤ 0.05 and fold-change ≥ 1.5; Figure 7(c); Table S1). Of these 588 HuR-dependent genes, 381 (65%) were found to be decreased in *Adipo-HuR^−/-^* mice, consistent with the traditional role of HuR as a mediator of increased RNA stability of target genes (Figure 7(c)) [9].

Gene ontology (GO) enrichment analysis of the HuR-dependent genes showed a significant enrichment of genes involved in ion transport, which represent 12 of the 43 total GO enriched terms (Figure 7(d); See Table S2 for a list of all significantly enriched GO terms). Furthermore, the expression of the majority (25/32; 78%) of significantly changed genes from the parent GO0006811 *Ion Transport* are decreased in *Adipo-HuR^−/-^* mice (Figure 7(e)). More specifically, genes (and GO groups) representing calcium transport were highly represented in this group (Figure 7(d)). Assessment of individual expression of significant genes within the most highly significant calcium ion transport GO from Figure 7(d), along with additional genes known to play a key role in sarcoplasmic calcium cycling, show a significant loss in expression in almost all of these genes upon HuR deletion (Figure 7(f)).

## Discussion

The results presented herein demonstrate that mice with an adipocyte-specific deletion of HuR are leaner and display increased energy expenditure compared to their wild-type control littermates. Upon closer examination of individual adipose depots, the most striking phenotype was within the BAT, where HuR deletion disrupted BAT architecture and resulted in decreased lipid droplet size. Functionally, this resulted in an impairment in adaptive thermogenesis in *Adipo-HuR^−/-^* mice that is independent of UCP1 expression. RNA-seq analysis suggests this may be due, at least in part, to decreased expression of HuR-dependent calcium cycling genes in *Adipo-HuR^−/-^* mice.

This work is the first to identify HuR as a mediator of thermogenesis, but is additionally significant as it is independent of UCP1-mediated thermogenesis. UCP1 plays a clear role in energy expenditure and obesity, and UCP1 overexpression in adipose tissue protects against diet-induced obesity [7]. Initial studies using UCP1 knockout mice showed that the loss of UCP1 yielded a surprising protection from obesity [8]. In fact, the impaired thermogenesis phenotype of the *Adipo-HuR^−/-^* mice is similar to that of *Ucp1^−/-^* mice first reported by Enerbäck et al [8]. Thus, we were surprised to find that HuR deletion has no effect on the expression levels of UCP1, especially given that UCP1 has been previously shown to be post-transcriptionally regulated via its 3ʹUTR [20] and HuR is an RNA binding protein that typically exerts post-transcriptional gene regulation via mRNA targeting at the 3ʹUTR [9]. The lean phenotype observed in *Ucp1^−/-^* mice was likely due to a compensatory increase in skeletal muscle shivering to generate heat in the absence of BAT-mediated thermogenesis, as later studies showed that UCP1 deletion promoted obesity when mice were housed at thermoneutral temperatures [21]. While the driving force behind the lean phenotype and increased energy expenditure of the *Adipo-HuR^−/-^* mice remains unclear, it is quite possible that compensatory increases in alternative, and perhaps less efficient, thermogenic pathways (shivering or non-shivering) needed to maintain basal core body temperature are driving increased energy expenditure.

This work demonstrates that HuR expression in adipocytes mediates energy expenditure at the organismal level and is required for acute adaptation to cold, independent of traditional UCP1-dependent thermogenesis. Mechanisms of non-shivering thermogenesis are most commonly attributed to the induction of UCP1 expression downstream of β_3_-adrenergic signalling, and have focused almost exclusively on UCP1, as it was thought as recently as 2001 that UCP1 was solely responsible for non-shivering thermogenesis [22]. However, *Ucp1^−/-^* mice have now been shown to still maintain thermogenic adaptation [23], and alternative pathways such as creatine kinase [24–26], calcium cycling [23,27–29], and fatty acid oxidation [30–32] have been identified as three primary mechanisms of UCP1-independent thermogenesis that occur through futile (non-ATP producing) substrate cycling [33]. Our results showing that acute pharmacological inhibition of HuR impairs thermogenesis suggest that HuR plays a direct role in the acute signalling response to thermogenesis and the observed deficiency in *Adipo-HuR^−/-^* mice is not simply a compensatory response. The presence of smaller lipid droplets in the BAT from mice lacking HuR is interesting and suggestive of a possible fuel shortage mechanism in BAT from *Adipo-HuR^−/-^* mice. However, it has been shown that lipolysis of lipid droplets within BAT is not required for thermogenesis [34] and, more recently, that lipid droplets within BAT are entirely dispensable for cold-induced thermogenesis [35].

Here, we employed unbiased RNA-seq analysis to compare transcriptome wide changes in gene expression in *Adipo-HuR^−/-^* compared to control mice. While we generally found no changes in traditional thermogenesis genes (Fig. S4), consistent with our observed lack of change in UCP1 protein levels, our results indicated a strong suppression of genes involved in ion transport, specifically calcium transport, upon HuR deletion. Mechanistically, this suggests that the impaired adaptive thermogenesis in *Adipo-HuR^−/-^* mice is likely due, at least in part, to a loss of calcium cycling in brown adipocytes. Indeed, many of the HuR-dependent genes whose expression is reduced in *Adipo-HuR^−/-^* mice are known to play a direct role in sarcoplasmic reticulum and cytoplasmic calcium cycling, including *slc8a3* (Na^+^/Ca^2+^ exchanger), *atp2a1* (SERCA ATPase), *ryr1 and ryr2* (ryanodine receptors), *sln* (sarcolipin), *trdn* (triadin), *casq* (calsequestrin), and *hrc* (histidine-rich calcium-binding protein). It is also interesting to note the critical importance of calcium cycling in cardiac myocytes given our previous work showing a role for HuR in cardiovascular disease [15,16], raising the possibility of mechanistic overlap for HuR-mediated gene networks in myocytes and adipocytes.

Given the unsuccessful translation of UCP1 targeting therapeutics to date [36–39], increasing effort has been directed at identifying UCP1-independent mediators of thermogenesis. Future work will further define the role of HuR-mediated thermogenesis within this field, but the continued delineation of the underlying mechanisms for the observations described herein will improve our understanding of BAT-mediated thermogenesis and metabolic homoeostasis.

## Supplementary Material

Supplemental Material
