# Supplementary material for: HuR expression in adipose tissue mediates energy expenditure and acute thermogenesis independent of UCP1 expression
Source: Adipocyte. 2020 Jul 25;9(1):336–46. doi: 10.1080/21623945.2020.1782021 (PMC7469577; doi:10.1080/21623945.2020.1782021)
Supplement: Supplemental Material [file KADI_A_1782021_SM7539.zip › Adipocyte Supp Figs ReSub.pdf]

**Figure S1.** HuR deletion in Adipo-HuR$^{-/-}$ mice is specific to adipose tissue.

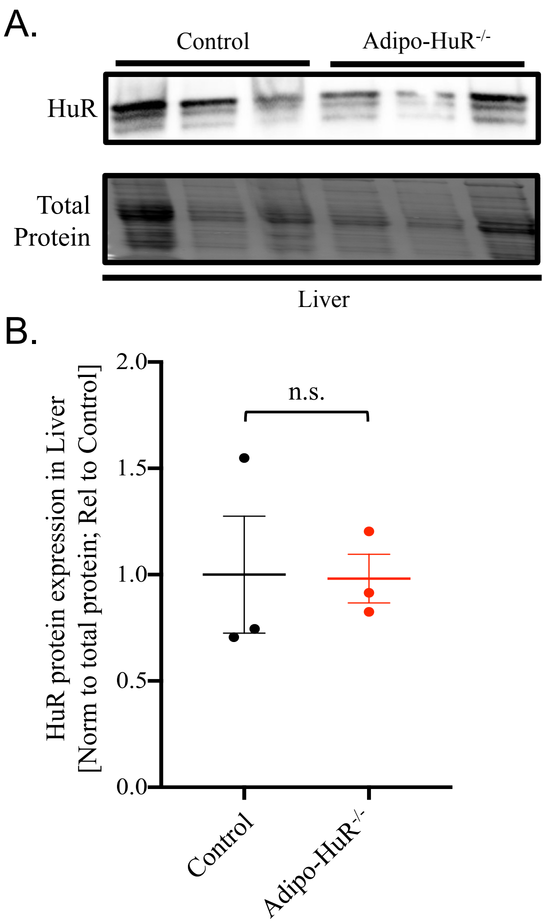

**Figure S2.** Decreased body mass in Adipo-HuR$^{-/-}$ mice is independent of developmental defects.

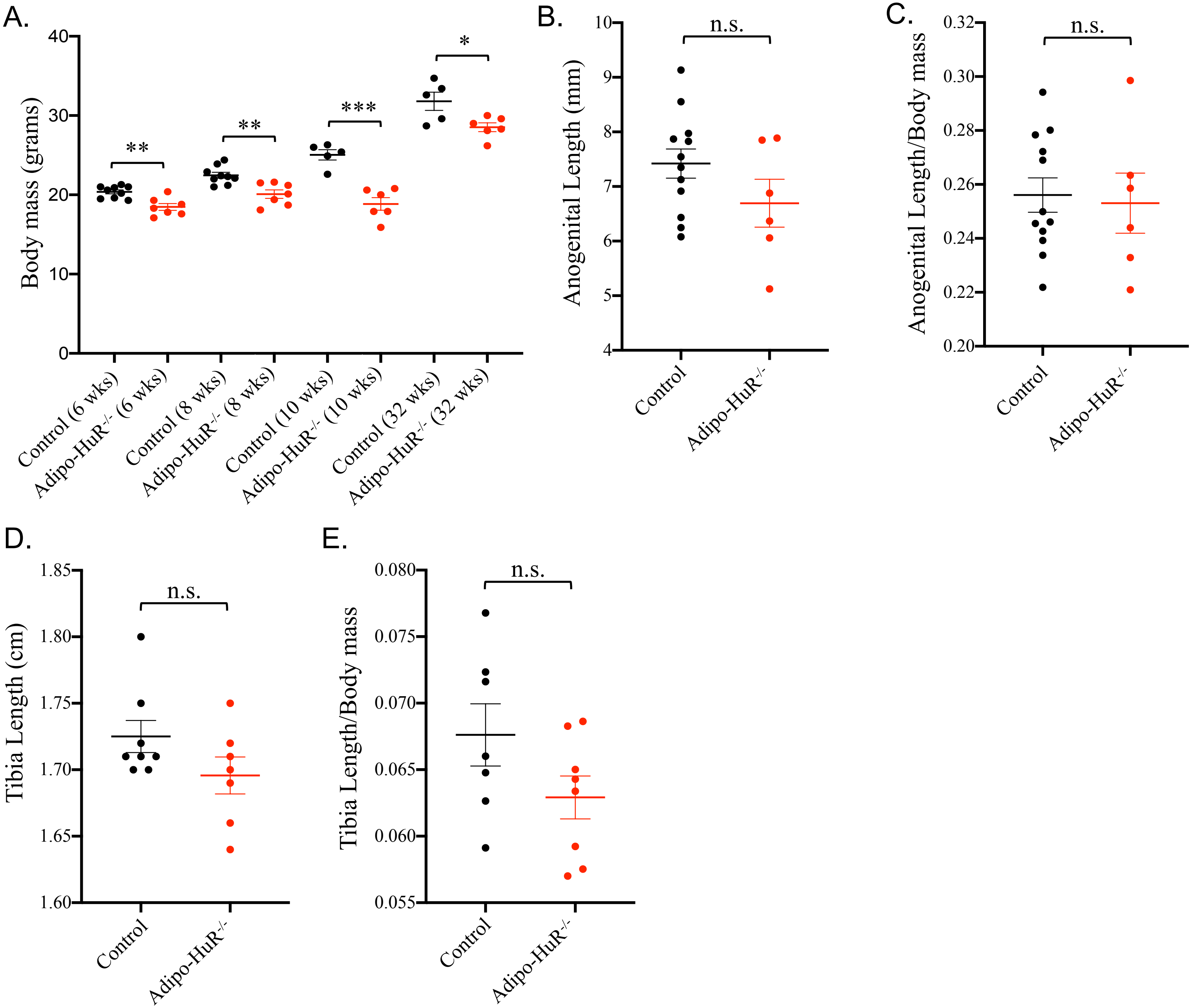

**Figure S3.** Energy expenditure is increased in obese Adipo- HuR$^{-/-}$ mice.

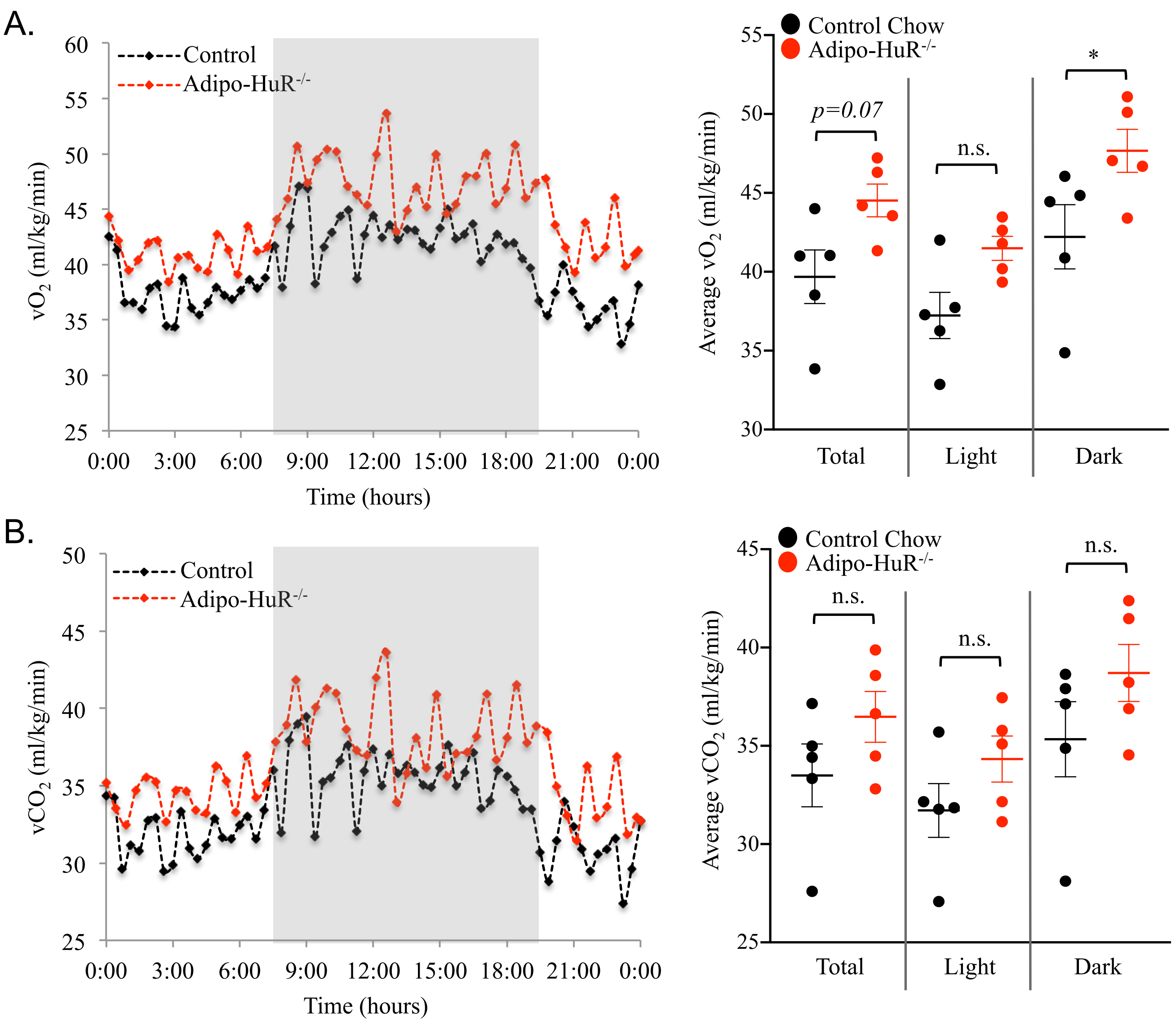

C.

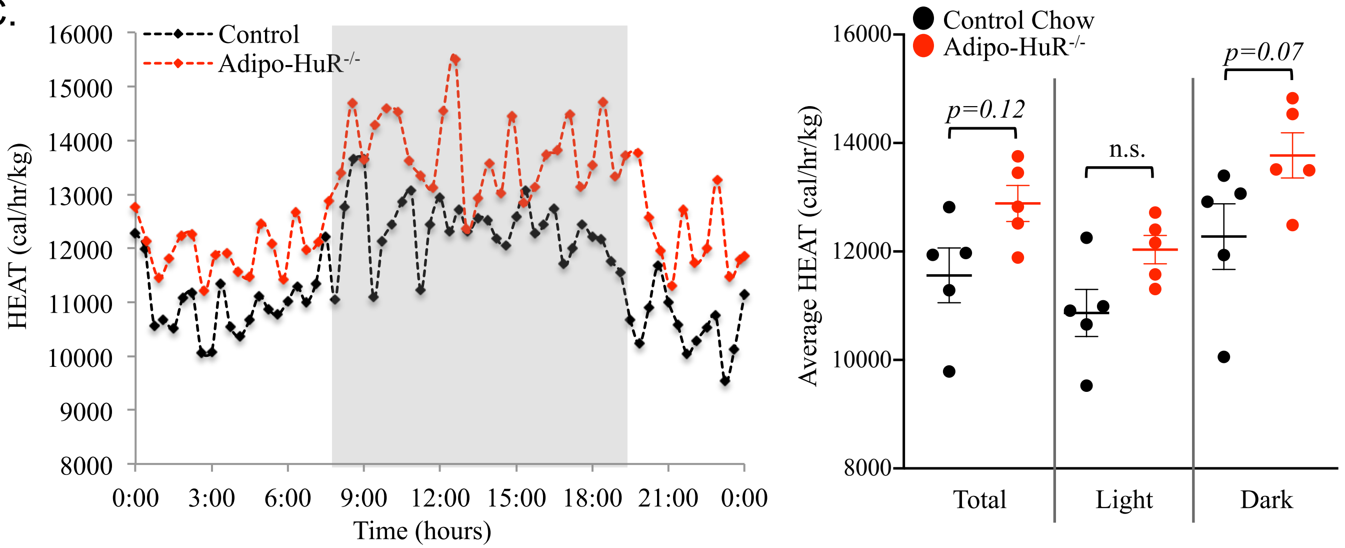

Control
Adipo-HuR-/-
HEAT (cal/hr/kg)
16000
15000
14000
13000
12000
11000
10000
9000
8000
0:00
3:00
6:00
9:00
12:00
15:00
18:00
21:00
0:00
Time (hours)
Control Chow
Adipo-HuR-/-
Average HEAT (cal/hr/kg)
16000
14000
12000
10000
8000
p=0.12
n.s.
p=0.07
Total
Light
Dark


D.

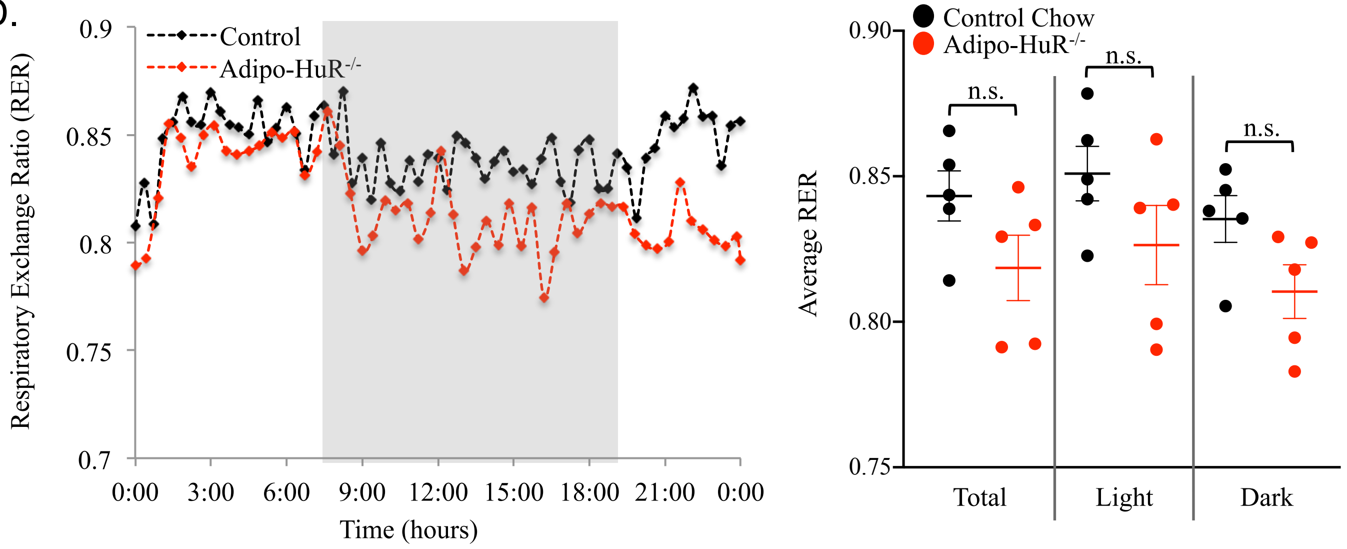

Control
Adipo-HuR-/-
Respiratory Exchange Ratio (RER)
0.9
0.85
0.8
0.75
0.7
0:00
3:00
6:00
9:00
12:00
15:00
18:00
21:00
0:00
Time (hours)
Control Chow
Adipo-HuR-/-
Average RER
0.90
0.85
0.80
0.75
n.s.
n.s.
n.s.
Total
Light
Dark

**Figure S4.** BAT from Adipo-HuR$^{-/-}$ mice shows no change in traditional thermogenesis genes.

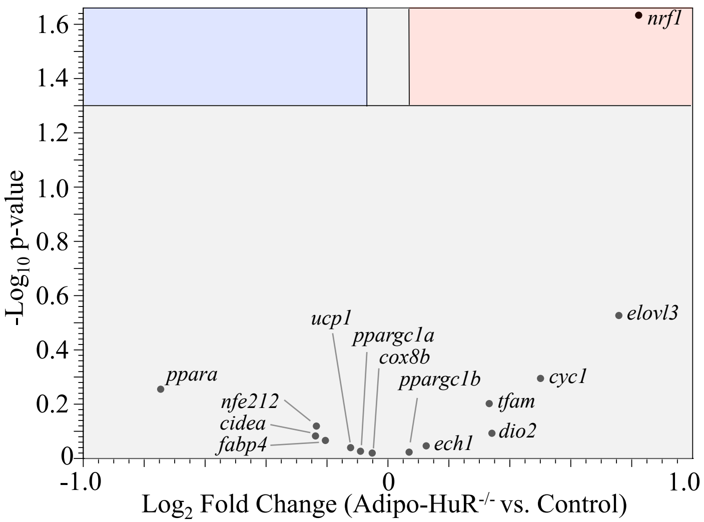

**Table S1.** All significant HuR-dependent gene expression changes in BAT.

*See attached excel file "Table S1"*

**Table S2.** All enriched GO groups among HuR-dependent gene expression in BAT.

| GO Term | Count | P-Value | Fold Enrichment |
|---|---|---|---|
| GO:0006810~transport | 61 | 0.049 | 1.26 |
| GO:0006811~ion transport | 32 | 2.16E-04 | 2.06 |
| GO:0006936~muscle contraction | 22 | 1.73E-20 | 16.51 |
| GO:0035556~intracellular signal transduction | 22 | 0.003 | 2.06 |
| GO:0007507~heart development | 21 | 2.41E-05 | 3.02 |
| GO:0016567~protein ubiquitination | 21 | 0.002 | 2.18 |
| GO:0007155~cell adhesion | 21 | 0.035 | 1.62 |
| GO:0055085~transmembrane transport | 20 | 0.004 | 2.06 |
| GO:0006816~calcium ion transport | 15 | 2.43E-05 | 3.99 |
| GO:0060048~cardiac muscle contraction | 14 | 2.35E-10 | 10.94 |
| GO:0034765~regulation of ion transmembrane transport | 14 | 6.50E-05 | 3.89 |
| GO:0003009~skeletal muscle contraction | 12 | 2.27E-11 | 17.31 |
| GO:0006937~regulation of muscle contraction | 11 | 1.36E-10 | 17.94 |
| GO:0045214~sarcomere organization | 11 | 4.34E-09 | 13.31 |
| GO:0006874~cellular calcium ion homeostasis | 11 | 3.29E-04 | 4.13 |
| GO:0007519~skeletal muscle tissue development | 10 | 2.07E-05 | 6.47 |
| GO:0070588~calcium ion transmembrane transport | 10 | 5.50E-04 | 4.26 |
| GO:0042391~regulation of membrane potential | 10 | 0.001 | 3.87 |
| GO:0009408~response to heat | 9 | 1.24E-04 | 5.92 |
| GO:0007517~muscle organ development | 9 | 2.54E-04 | 5.36 |
| GO:0051592~response to calcium ion | 9 | 4.31E-04 | 4.97 |
| GO:0051726~regulation of cell cycle | 9 | 0.010 | 3.01 |
| GO:0007015~actin filament organization | 8 | 0.007 | 3.62 |
| GO:0006941~striated muscle contraction | 7 | 8.60E-07 | 18.76 |
| GO:0030239~myofibril assembly | 7 | 1.40E-06 | 17.51 |
| GO:0048741~skeletal muscle fiber development | 7 | 6.32E-05 | 9.73 |
| GO:0008104~protein localization | 7 | 0.019 | 3.32 |
| GO:0071805~potassium ion transmembrane transport | 7 | 0.033 | 2.92 |
| GO:0055008~cardiac muscle tissue morphogenesis | 6 | 4.47E-05 | 14.07 |
| GO:0055010~ventricular cardiac muscle tissue morphogenesis | 6 | 7.73E-04 | 8.04 |
| GO:0002027~regulation of heart rate | 6 | 0.002 | 6.25 |
| GO:0035914~skeletal muscle cell differentiation | 6 | 0.015 | 4.09 |
| GO:0010033~response to organic substance | 6 | 0.029 | 3.46 |
| GO:0051289~protein homotetramerization | 6 | 0.047 | 3.04 |
| GO:0051384~response to glucocorticoid | 6 | 0.049 | 3.00 |
| GO:0014883~transition between fast and slow fiber | 5 | 9.14E-05 | 18.76 |
| GO:0055003~cardiac myofibril assembly | 5 | 2.07E-04 | 15.63 |
| GO:0045662~negative regulation of myoblast differentiation | 5 | 0.005 | 7.21 |
| GO:0007274~neuromuscular synaptic transmission | 5 | 0.005 | 6.95 |
| GO:0005977~glycogen metabolic process | 5 | 0.020 | 4.81 |
| GO:0090002~establishment of protein localization to plasma membrane | 5 | 0.027 | 4.36 |
| GO:0051496~positive regulation of stress fiber assembly | 5 | 0.029 | 4.26 |
| GO:0034220~ion transmembrane transport | 5 | 0.036 | 3.99 |
