# Supplementary material for: HuR expression in adipose tissue mediates energy expenditure and acute thermogenesis independent of UCP1 expression
Source: Adipocyte. 2020 Jul 25;9(1):336–46. doi: 10.1080/21623945.2020.1782021 (PMC7469577; doi:10.1080/21623945.2020.1782021)
Supplement: Supplemental Material [file KADI_A_1782021_SM7539.zip › Figure Supp caption.docx]

**Figure S1. HuR deletion in Adipo-HuR^-/-^ mice is specific to adipose tissue.** (**A**) Western blot showing relative HuR protein expression in liver tissue from control and Adipo-HuR^-/-^ mice. (**B**) Quantification of the blot in (**A**) shows no significant difference in HuR expression between control and Adipo-HuR^-/-^ mice.

**Figure S2. Decreased body mass in Adipo-HuR^-/-^ mice is independent of developmental defects.** (**A**) Body mass assessment of control and Adipo-HuR^-/-^ mice at 6, 8, 10, and 32 weeks old shows difference in body mass from time of weaning. (**B, C**) Anogenital length measurement shows no difference between control and Adipo-HuR^-/-^ mice when presented as raw length or normalized to body mass, respectively. (**D, E**) Tibia length measurement shows no difference between control and Adipo-HuR^-/-^ mice when presented as raw length or normalized to body mass, respectively.

**Figure S3. Energy expenditure is increased in obese Adipo-HuR^-/-^ mice.** Daily oxygen consumption (vO_2_) (**A**) and CO_2_ production (vCO_2_) (**B**) were measured using the Oxymax system. Total energy expenditure (HEAT) (**C**) and respiratory exchange ratio (RER) (**D**) were then determined based on vO_2_ and vCO_2_ values. Shaded area represents dark housing cycle and data is represented as both rolling daily average (left) and total, light, and dark averages (right). n ≥ 4 per group. **P* ≥ 0.05.

**Figure S4. BAT from Adipo-HuR^-/-^ mice shows no change in traditional thermogenesis genes.**

Volcano plot of fold change and p-values shows no change (with the exception of *nrf1*) in traditional thermogenesis genes in BAT from Adipo-HuR^-/-^ mice. Blue and red regions represent significantly down and up-regulated genes, respectively.

**Table S1. All significant HuR-dependent gene expression changes in BAT.**

**Table S2. All enriched GO groups among HuR-dependent gene expression in BAT.**
